# Study on Physicochemical Properties, Antioxidant Activity and Flavor Quality in the Fermentation of a Plant-Based Beverage by Different Lactic Acid Bacteria

**DOI:** 10.3390/foods14213761

**Published:** 2025-11-02

**Authors:** Liu Yang, Yifan Zhao, Yingzhuo Zhou, Qian Zhao, Shaohua Yuan, Chen Ma, Li Dong, Yinghua Luo, Xiaosong Hu, Fang Chen, Daotong Li

**Affiliations:** 1 Key Laboratory of Fruits and Vegetables Processing, National Engineering Research Center for Fruits and Vegetables Processing, College of Food Science and Nutritional Engineering, Ministry of Agriculture, China Agricultural University, No.17, Qinghua East Road, Haidian District, Beijing 100083, China; liu_yang@cau.edu.cn (L.Y.); 17810768001@163.com (Y.Z.); 18321782631@163.com (Y.Z.); 18510231522@163.com (Q.Z.); s20243061279@cau.edu.cn (S.Y.); machen21@cau.edu.cn (C.M.); li_dong127@163.com (L.D.); luoyinghua@cau.edu.cn (Y.L.); huxiaos@263.net (X.H.); chenfangch@sina.com.cn (F.C.); 2 Engineering Research Centre for Fruits and Vegetables Processing, Ministry of Education, China Agricultural University, No.17, Qinghua East Road, Haidian District, Beijing 100083, China

**Keywords:** medicinal and edible plant-based beverage, lactic acid bacteria, physicochemical properties, HS-SPME-GC-MS, flavor profiles

## Abstract

This study investigated the effects of three different lactic acid bacteria (LAB) strains (*Limosilactobacillus fermentum* 14, *Limosilactobacillus reuteri* 18, and *Lactiplantibacillus plantarum* CAU808) on the nutrient components, bioactivity, and flavor profiles of a medicinal and edible homologous (MEH) plant-based beverage (QJ). Results demonstrated that QJ served as an excellent substrate for LAB growth, with viable counts of all three LAB exceeding 8.5 log CFU/mL after fermentation. Fermentation significantly reduced soluble sugar contents while increasing organic acids levels. A slight enhancement in ABTS radical scavenging capacity was also observed. Electronic tongue (E-tongue) analysis revealed that LAB fermentation markedly decreased bitterness and enhanced sourness, sweetness, and umami, thereby improving the overall taste profile. Furthermore, electronic nose (E-nose) and HS-SPME-GC-MS analyses indicated distinct alterations in odor characteristics post-fermentation. A total of 87 volatile compounds were identified, with alcohols constituting the predominant group. Compared to the other two strains, *Lactiplantibacillus plantarum* CAU808 demonstrated superior fermentation performance and more favorable flavor characteristics. These findings provide a theoretical basis for utilizing LAB fermentation to optimize the flavor of MEH plant-based beverages.

## 1. Introduction

The global rise in the incidence of chronic diseases such as type 2 diabetes mellitus, hypertension, and dyslipidemia has driven consumers to choose functional products with health-promoting properties [1]. Rooted in traditional Chinese medicine, the medicinal and edible homologous (MEH) concept emphasizes the intrinsic overlap between nutritional and functional properties of plants. In recent years, MEH plants have emerged as pivotal resources of functional beverages. Botanically, these plants contain bioactive compounds that confer nutritional and pharmacological benefits. Common examples include goji berry (*Lycium barbarum* L.) and chrysanthemum (*Chrysanthemi Flos*) [2]. Modern pharmacological studies have validated their potent antioxidant, anti-inflammatory, and antidiabetic activities [3,4,5]. Despite their health benefits, MEH plant-based food products face challenges in masking undesirable flavor, which limits consumer acceptance and preference. Therefore, it is necessary to explore advanced processing technology for maintaining biological properties while improving flavor characteristics.

Probiotic lactic acid bacteria (LAB) fermentation, a simple and promising food processing technology, has gained prominence in plant-based functional beverage development due to its ability to enhance bioactive compound release, modify flavor profiles, and extend shelf life [6,7]. Recent advancements highlight LAB fermentation as a strategy to amplify both functional and sensory attributes of the functional beverage. For instance, *Lactiplantibacillus plantarum* fermentation of elderberry (*Sambucus nigra* L.) juice increased volatile compounds, improving flavor complexity and antioxidant capacity [8]. Similarly, *Weissella cibaria* 64 and *Leuconostoc mesenteroides* 12b enhanced bioactive compounds in tropical fruit juices by boosting exopolysaccharide production and antioxidant levels [9]. LAB fermentation of ginkgo (*Ginkgo biloba* L.) seed juice reduced toxic ginkgolic acid content while enriching terpene lactones, significantly improving antioxidant and antimicrobial activities [10]. Additionally, LAB metabolites may benefit gut microbiota and immune modulation [11]. However, the application potential of LAB fermentation in the development of MEH plant-based beverages and its effect on the physicochemical properties and flavor quality are not yet fully understood. The strain-specific metabolic activities of LAB can unpredictably alter the flavor profiles of fermented products. For instance, Liang et al. [12] observed that *Lactiplantibacillus plantarum* had a more significant influence on volatile compounds in sweet potato juice compared to four other LAB strains. Furthermore, Yuan and her colleagues demonstrated that genomic variations among different strains are responsible for these divergent flavor characteristics [13].

In this study, we systematically evaluated the effects of different LAB strains on the physicochemical properties, biological activities, and flavor characteristics of a MEH plant-based beverage. By comparing the effects of different LAB strains on the same MEH substrate, we aimed to (1) quantify fermentation-induced physicochemical changes; (2) assess alterations in antioxidant capacity; and (3) characterize flavor profiles using E-tongue/nose systems and volatile compound analysis. This study provides new insights into the application of LAB fermentation in development of MEH plant-based beverages.

## 2. Materials and Methods

### 2.1. Materials

The strains *Limosilactobacillus fermentum* 14 (Lf14), *Limosilactobacillus reuteri* 18 (Lr18), and *Lactiplantibacillus plantarum* CAU808 (Lp808) were preserved at the National Engineering Research Center for Fruits and Vegetables Processing, China Agricultural University (Beijing, China). The 16S rRNA sequencing results of the strains have been uploaded to National Center for Biotechnology Information (NCBI), and the accession numbers are as follows: Lf14 (SUB15725043 seq1 PX470201), Lr18 (SUB15725043 seq1 PX470202), Lp808 (SUB15725043 seq1 PX470203). Dried chrysanthemum (*Chrysanthemum morifolium*; batch 2411011), goji berries (*Lycium barbarum*; batch 2411012), wine-processed polygonatum (*Polygonatum kingianum*; batch 2411014), codonopsis root (*Codonopsis pilosula*; batch 2411013), roasted cassia seeds (*Cassia obtusifolia*; batch 2409011), and rose flowers (*Rosa rugosa*; batch 2410011) were purchased from Hebei Baicaokangshen Pharmaceutical Co., Ltd. (Hengshui, China). Organic acid standards (purity ≥ 98%) were obtained from Shanghai Yuanye Bio-Technology Co., Ltd. (Shanghai, China), and 2-octanol standard (purity ≥ 99.5%) was sourced from Shanghai Macklin Biochemical Technology Co., Ltd. (Shanghai, China). All chemicals used in the experiments were of analytical grade unless explicitly stated otherwise.

### 2.2. Preparation of LAB Strains

De Man, Rogosa and Sharpe (MRS) broth was used for the cultivation of LAB. Strains *Limosilactobacillus fermentum* 14 (Lf14), *Limosilactobacillus reuteri* 18 (Lr18), and *Lactiplantibacillus plantarum* CAU808 (Lp808) were revived and subcultured twice to the logarithmic growth phase. Cell suspensions were centrifuged at 5000 rpm for 5 min, followed by supernatant removal. The pellets were washed three times with sterile saline and resuspended in saline for subsequent use.

### 2.3. Preparation and Fermentation of the MEH Plant-Based Beverage (QJ)

QJ formulation and preparation protocol were provided by Xiyuan Hospital, China Academy of Chinese Medical Sciences (Beijing, China). The specific steps were as follows: 15 g chrysanthemum, 15 g goji berries, 10 g wine-processed polygonatum, 15 g codonopsis root, 6 g roasted cassia seeds and 6 g rose flowers were weighed, rinsed thoroughly, and decocted with four times their weight of water for 12 h to obtain the aqueous QJ extract. The pH of the aqueous QJ extract was 4.75 ± 1.00, and the soluble sugar content was 28.0 ± 2.0 mg/mL. The extract was heated in a water bath at 85 °C for 15 min, then rapidly cooled to 4–5 °C. Fermentation was carried out in 50 mL sterile centrifuge tubes, with a working volume of 30 mL of QJ medium and a headspace liquid ratio of 2:3. Under aseptic conditions, the activated bacterial inoculate was inoculated into QJ at a vaccination rate of 5%, and then incubated with shaking at 37 °Cand 200 rpm for 48 h in a sealed environment (without the use of oxygen scavengers). The fermentation process was terminated at 48 h based on preliminary experiments and established protocols for LAB fermentation.

### 2.4. Bacterial Enumeration and pH

The viable count of LAB in fermented samples was determined using the plate count method and expressed as log CFU/mL [14].

Changes in pH during fermentation were monitored using a calibrated PB-10 pH meter (Sartorius, Göttingen, Germany).

### 2.5. Nutrient Composition

#### 2.5.1. Soluble Sugar

The soluble sugar content in samples was quantified using the anthrone-sulfuric acid colorimetric method with slight modifications based on the protocol by Tang et al. [15]. Fresh samples were homogenized in distilled water (1:10 *v*/*v*), heated in a boiling water bath for 10 min, and cooled. The mixture was centrifuged at 8000× *g* for 10 min at room temperature, and the supernatant was collected and diluted to a final volume of 10 mL with distilled water. The test solution was mixed with anthrone reagent and concentrated sulfuric acid in a centrifuge tube. The mixture was incubated in a hot water bath (95 °C) for 10 min, cooled to room temperature, and absorbance was measured at 620 nm using a microplate reader (SpectraMax iD5, Molecular Devices, California, CA, USA). Soluble sugar content was calculated against a glucose standard curve (0.0125–0.3 mg/mL) and expressed as mg/mL.

#### 2.5.2. Total Protein

The total protein content in samples was determined using the Biuret method with minor modifications as described by Liu and Pan [16]. Briefly, fresh samples were homogenized in distilled water (1:5 *v*/*v*) in an ice bath and centrifuged at 8000× *g* for 10 min at 4 °C. The supernatant was collected for analysis. The test solution was mixed with Biuret reagent and incubated at room temperature for 15 min. Absorbance of the resulting solution was measured at 540 nm using a microplate reader (SpectraMax iD5, Molecular Devices, California, USA). The total protein concentration was calculated according to the concentration and absorbance of bovine serum albumin (BSA) standard solution, and the results were expressed in mg/mL.

#### 2.5.3. Total Amino Acid

The total amino acid content of samples was determined using an amino acid assay kit (Solarbio Science & Technology Co., Ltd., Beijing, China) according to the manufacturer’s instructions. Briefly, fresh samples were processed as specified in the protocol, and absorbance was measured at 570 nm using a microplate reader (SpectraMax iD5, Molecular Devices, California, USA). The total amino acid concentration in the sample was calculated according to the concentration of glutamic acid standard solution and absorbance, and the results were expressed in μmol/mL.

#### 2.5.4. Total Phenol Content (TPC)

TPC of samples was determined using the Folin–Ciocalteu (FC) colorimetric method with minor modifications based on the protocol described by Wang et al. [17]. Briefly, total phenol was extracted from the samples with 60% (*v*/*v*) ethanol. An amount of 50 μL of the extract was thoroughly mixed with 50 μL of FC reagent and incubated at 25 °C for 10 min. Subsequently, 200 μL of 7.5% (*w*/*v*) Na_2_CO_3_ solution was added to the mixture and vortexed. The reaction proceeded in the dark at room temperature for 30 min. Absorbance of the resulting solution was measured at 765 nm using a microplate reader (SpectraMax iD5, Molecular Devices, California, USA). A standard curve was established using gallic acid (4.9–156.25 μg/mL), and TPC was expressed as milligrams of gallic acid equivalents per milliliter of sample (mg GAE/mL).

#### 2.5.5. Total Flavonoid Content (TFC)

TFC of samples was determined by using the method described by Meng et al. [18] with modifications. Total flavonoid was extracted from the samples with 60% (*v*/*v*) ethanol. A quantity of 1 mL of the extract was mixed with 0.5 mL of 5% (*v*/*v*) NaNO_2_ and reacted at ambient temperature for 5 min. Subsequently, 1 mL of 10% (*w*/*v*) AlCl_3_ was added, and the mixture was incubated in the dark for 5 min. Finally, 4 mL of 4% (*w*/*v*) NaOH was added, and the solution was adjusted to a final volume of 10 mL with 60% (*v*/*v*) ethanol, followed by incubation in the dark for 15 min. Absorbance was measured at 510 nm using a microplate reader (SpectraMax iD5, Molecular Devices, California, USA). A standard curve was constructed using rutin (concentration range: 0.2–1.25 mg/L), and TFC was expressed as milligrams of rutin equivalents per milliliter of sample (mg RE/mL).

### 2.6. Organic Acids

Organic acids in the samples were analyzed using high-performance liquid chromatography (HPLC) equipped with a diode array detector (Agilent 1200 Infinity, Agilent, California, USA). Samples were centrifuged at 10,000 rpm for 10 min and filtered through a 0.22 μm membrane. Separation was performed on an Alphasil VC-C18 column (150 × 4.6 mm, 3.5 μm; Acchrom Tech, Beijing, China) under isocratic elution with a mobile phase of 0.1% H3PO4 and methanol (97.5:2.5). The column temperature was maintained at 30 °C, with a flow rate of 1.0 mL/min. Detection wavelength and injection volume were set to 210 nm and 10 μL, respectively. Organic acids in the samples were identified and quantified by comparing retention times (RT) and regression equations with a mixed standard solution containing eight organic acids: malic acid, citric acid, lactic acid, acetic acid, fumaric acid, succinic acid, maleic acid, and oxalic acid.

### 2.7. Antioxidant Activity

#### 2.7.1. Ferric Reducing Antioxidant Power (FRAP) Assay

The antioxidant capacity of samples was evaluated using the FRAP assay, adapted from a previously reported method [19] with minor modifications. Briefly, the FRAP reagent was prepared by mixing 0.1 M acetate buffer (pH 3.6), 10 mM 2,4,6-tripyridyl-s-triazine (TPTZ) solution, and 20 mM FeCl_3_ solution at a ratio of 10:1:1. 0.1 mL of the sample extract was mixed with 2.9 mL of FRAP reagent and incubated in the dark at ambient temperature for 15 min. Absorbance of the reaction mixture was measured at 593 nm using a microplate reader (SpectraMax iD5, Molecular Devices, California, CA, USA). A calibration curve was constructed with freshly prepared FeSO_4_ standards (0–150 μM), and the FRAP was expressed as micromoles of Fe^2+^ equivalents per milliliter of sample (μmol Fe^2+^/mL).

#### 2.7.2. ABTS Radical Scavenging Capacity

The ABTS radical scavenging activity of the samples was evaluated according to a previously reported method [19] with minor modifications. Briefly, 0.1 mL of the sample was mixed with 1.4 mL of distilled water and centrifuged at 10,000 rpm and 4 °C for 10 min. The ABTS working stock solution was prepared by mixing 3.84 g/L ABTS and 0.66 g/L K_2_S_2_O_8_ at a 1:1 ratio, followed by incubation in the dark at 25 °C for 16 h. The solution was then diluted with phosphate-buffered saline (PBS, pH 7.4) to achieve an absorbance of 0.70 ± 0.02 at 734 nm. Subsequently, 0.1 mL of the sample supernatant was mixed with 9.9 mL of the ABTS working solution and incubated in the dark for 15 min. Absorbance was measured at 734 nm using a microplate reader (SpectraMax iD5, Molecular Devices, California, CA, USA). The scavenging activity (%) was calculated using the formula:

(1) $$\%ABTS=\left[\frac{A_{blank}-\left(A_{sample}-A_{sample-blank}\right)}{A_{blank}}\right]\times 100,$$

where $A_{blank}$ is the absorbance after the reaction of distilled water and ABTS working liquid; $A_{sample}$ is the absorbance of sample supernatant reacting with ABTS working liquid; $A_{sample-blank}$ is the absorbance of sample supernatant after reaction with distilled water.

#### 2.7.3. DPPH Radical Scavenging Capacity

The DPPH radical scavenging capacity of samples was evaluated according to the previously reported method [19] with minor modifications. Briefly, 0.1 mL of the sample was mixed with 1.4 mL PBS (pH 7.4) and centrifuged at 10,000 rpm for 10 min at room temperature. Subsequently, 0.1 mL of the supernatant was reacted with 4.9 mL of 20 mg/L DPPH solution and incubated in darkness for 30 min. The absorbance was measured at 517 nm using a microplate reader (SpectraMax iD5, Molecular Devices, California, CA, USA). The scavenging rate (%) was calculated using the formula:

(2) $$\%DPPH=\left[\frac{A_{blank}-\left(A_{sample}-A_{sample-blank}\right)}{A_{blank}}\right]\times 100,$$

where $A_{blank}$ represents the absorbance of PBS mixed with DPPH solution; $A_{sample}$ denotes the absorbance of the sample supernatant reacted with DPPH solution; $A_{sample-blank}$ corresponds to the absorbance of the sample supernatant mixed with anhydrous ethanol.

### 2.8. Bionic Sensory

#### 2.8.1. Electronic Tongue (E-Tongue)

Taste profiles were evaluated using an E-tongue (Astree, Alpha MOS, Toulouse, France). The E-tongue has seven sensors corresponding to different tastes: AHS (sourness), CTS (saltiness), NMS (umami), ANS (sweetness), SCS (bitterness), PKS (compound taste), CPS (compound taste). Samples were homogenized, diluted 30-fold with deionized water, and filtered through qualitative filter paper. Aliquots (25 mL) of the filtrate were transferred to E-tongue specific cups and loaded into the sampler. The system assessed taste intensity rankings for sour, sweet, bitter, salty, and umami.

#### 2.8.2. Electronic Nose (E-Nose)

Analysis was performed using an ultra-fast gas chromatography E-nose (Heracles Neo, Alpha MOS, Toulouse, France). Samples were homogenized, and 4.00 g aliquots were accurately weighed into headspace vials. Vials were sealed with PTFE septa and loaded into the automatic sampler for analysis. Data processing and compound identification were conducted using the instrument’s software with reference to the AroChemBase database.

### 2.9. Volatile Component

#### 2.9.1. Volatile Compounds

The volatile compounds in fermented liquids were analyzed by headspace solid-phase microextraction–mass spectrometry coupled with gas chromatography–mass spectrometry (HS-SPME-GC-MS) following the method of Yuan et al. [13] with modifications. Briefly, 5.0 g sample mixed with 1.5 g NaCl in a 20 mL headspace vial was spiked with 10 μL 2-octanol internal standard solution (8.22 × 10^−5^ g/mL). After equilibration at 45 °C for 10 min with intermittent shaking (20 s on/2 s off), volatile components were extracted using a 50/30 μm DVB/CAR/PDMS fiber via HS-SPME at 45 °C for 40 min. GC-MS analysis was performed on a system equipped with a DB-WAX column under helium carrier gas (1.0 mL/min). The temperature program initiated at 35 °C (2 min hold), ramped to 45 °C at 2 °C/min, then to 130 °C at 5 °C/min, and finally to 225 °C at 10 °C/min (5 min hold). Electron ionization (70 eV) was applied with ion source and quadrupole temperatures at 230 °C and 150 °C, respectively. Full-scan mode (35–550 *m*/*z*) was used for detection.

The identification of volatile compounds was performed by comparing their mass spectra with those in the NIST Mass Spectral Library (NIST 17) and by comparing their Linear Retention Indices (LRI). The match criteria for the NIST library search were a similarity score ≥ 85. In the LRI method, under the same extraction and analysis conditions, 1 μL of a mixed n-alkane standard solution (C7–C30) was injected in liquid mode. The retention times (RT) of the n-alkanes were used to calculate the LRI of the compounds in the samples according to the following formula:

(3) $$LRI=100N+100n(t_{RA}-t_{RN})/(t_{R(N+n)}-t_{RN})$$

where N is the carbon number of the n-alkane eluting immediately before the target compound, n is the difference in carbon number between the n-alkanes eluting immediately before and after the target compound, $t_{RA}$ is the retention time of the target compound, $t_{RN}$ is the retention time of the n-alkane eluting immediately before the target compound, and $t_{R(N+n)}$ is the retention time of the n-alkane eluting immediately after the target compound. The calculated LRI of a compound was compared with its reference LRI from the NIST Chemistry WebBook (https://webbook.nist.gov/chemistry/, accessed on 26 October 2025), and a difference ≤ 20 was used as the identification criterion.

The quantification of volatile compounds was carried out using an internal standard method with correction factors, calculated using the following formula:

(4) $$C=C_{S}\times f\times A/A_{S}$$

where C and A represent the concentration and peak area of the target compound, respectively, $C_{S}$ and $A_{S}$ represent the concentration and peak area of the internal standard, respectively, and *f* is the correction factor for the target compound. The correction factor f was determined from the ratio of the peak areas of the quantifier ions of the target compound standard and the internal standard.

#### 2.9.2. Odor Activity Value (OAV)

Quantification utilized peak area ratios relative to internal standard. Odor activity values (OAV) were calculated as compound concentration divided by its odor threshold [20].

### 2.10. Statistical Analysis

Data were analyzed using SPSS 27.0 (SPSS Inc., Chicago, IL, USA). One-way ANOVA with Duncan’s multiple range test (*p* < 0.05) was applied to determine significant differences. All experiments were conducted in triplicate, with results expressed as mean ± standard deviation (SD). The GraphPad Prism software (version 10.4.1) (GraphPad Software, San Diego, CA, USA) was used to plot data.

## 3. Results and Discussion

### 3.1. Analysis of Bacterial Counts and pH

**Figure 1:**
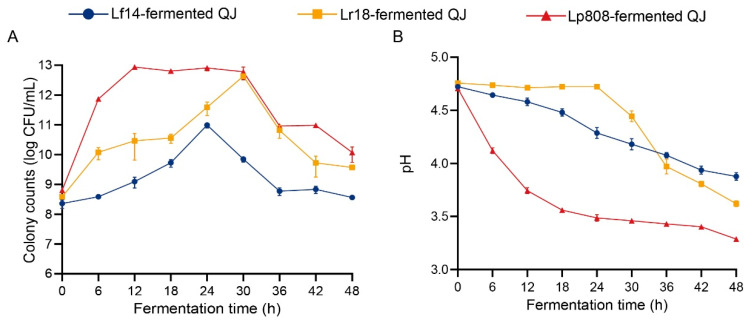
Changes in the bacterial colony counts (**A**) and pH (**B**) of QJ during three LAB fermentation.

During fermentation, bacterial enumeration and pH dynamics serve as critical monitoring parameters, reflecting the biological state of the fermentation system and metabolic environment, respectively [21]. Bacterial counts directly indicate bacterial proliferation and are essential for evaluating fermentation progress and probiotic effect of LAB [22]. Three LAB strains exhibited distinct growth patterns during the 48 h fermentation (Figure 1A). All strains demonstrated sustained viability throughout fermentation, maintaining final viable bacterial counts exceeding 8.50 ± 0.10 log CFU/mL. Lp808 exhibited the fastest growth rate in QJ, achieving a maximum viable count of 12.94 ± 0.03 log CFU/mL at 12 h. In contrast, Lr18 displayed delayed growth initiation, requiring 30 h to reach its peak concentration of 12.63 ± 0.06 log CFU/mL. Lf14 maintained consistently lower growth rates throughout the process that was inferior to other strains. These results indicated that the nutrients in QJ could meet the basic growth requirements of all three LAB strains particularly Lp808. This aligns with previous reports highlighting the adaptability of LAB to plant substrates, particularly *Lactiplantibacillus plantarum*, which is recognized for its superior metabolic versatility in utilizing plant-derived nutrients [23,24,25].

The pH variation reflects the metabolic activity of microorganisms during fermentation [26]. We observed strain-specific correlations between pH trends and bacterial growth characteristics (Figure 1B). Among the three strains, Lp808 demonstrated rapid acidification capacity, with QJ fermented using this strain reaching the lowest final pH of 3.29 ± 0.01. Lr18 exhibited moderate acid production, resulting in a terminal pH of 3.62 ± 0.03 in QJ, while Lf14 showed minimal pH reduction. These differences likely arose from strain-specific lactic acid production and other organic acids, which were able to inhibit the growth of competing microbiota and extend product shelf life [27,28]. The superior acidification capacity of Lp808 highlights its high metabolic efficiency in this fermentation system compared to the other tested strains.

### 3.2. Analysis of Nutrient Composition

#### 3.2.1. Soluble Sugar Analysis

**Figure 2:**
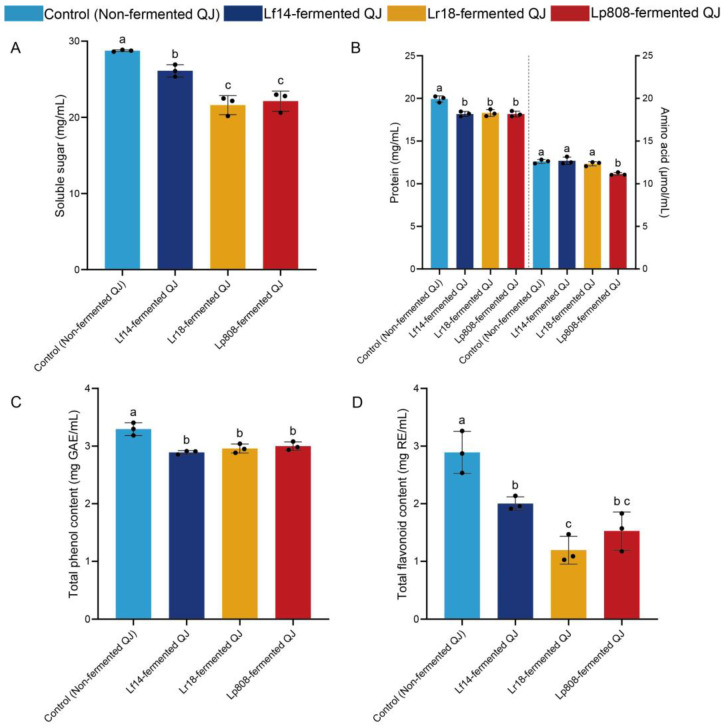
Changes in the soluble sugar (**A**), total protein and amino acid (**B**), total phenol content (**C**) and total flavonoid content (**D**) of control (non-fermented QJ) and all three LAB-fermented QJ. Letters were used to indicate statistical significance between samples (*p* < 0.05) based on one-way ANOVA.

Monitoring soluble sugars during fermentation is crucial as they serve as primary carbon sources for LAB growth and metabolic activities. As shown in Figure 2A and summarized in Table S1, the type of LAB fermentation had an extremely significant and substantial impact on the soluble sugar content of the QJ beverage (*p* < 0.0001), decreasing from 28.75 ± 0.12 to 26.11 ± 0.80, 21.61 ± 1.25, and 22.13 ± 1.32 mg/mL by the fermentation using the Lf14, Lr18 and Lp808 strains, respectively. The effect size was remarkably large (η^2^ = 0.929), indicating that the fermentation treatment accounted for over 92% of the variance in the data. The reduction likely resulted from microbial consumption for energy production and conversion to organic acids, consistent with typical LAB fermentation patterns [29]. Strain-specific variations in sugar utilization efficiency were observed, with Lr18 showing the highest consumption rate (24.8% reduction), potentially due to differential expression of glycosidase enzymes or preferential substrate utilization strategies among strains [30]. These differences highlight the importance of strain selection for targeted metabolic outcomes in fermentation processes.

#### 3.2.2. Total Protein and Amino Acid Content Analysis

LAB strain fermentation altered total protein and amino acid contents in QJ. Protein monitoring is essential as LAB proteolytic systems modify bioactive peptide availability [31]. For total protein, all three fermented groups showed significant and comparable reductions compared to the control (non-fermented QJ) (*p* = 0.0006, η^2^ = 0.873), corresponding to a decrease of 8.48–8.83% (Figure 2B, Table S1). No significant differences were observed among the three fermented groups themselves (*p* > 0.97). This universal decrease was likely due to the strains consuming proteins to meet their survival demands during fermentation [32]. In contrast, the changes in total amino acid contents exhibited significant strain-specific variations (Figure 2B, Table S1), reflecting the balance between protein degradation and microbial utilization [33]. Compared to control (non-fermented QJ), there were no significant change in the total amino acid contents of Lf14 and Lr18-fermented QJ (*p* = 0.985 and *p* = 0.658, respectively), which is likely maintained by the limited catabolism and compensatory synthesis of Lf14 and Lr18. However, Lp808-fermented QJ showed a significant reduction in total amino acids compared to the control, Lf14-, and Lr18-fermented QJ groups (*p* < 0.01), indicating that Lp808 utilized more amino acids or produced more secondary metabolites during growth. These functional variations may stem from differences in protease specificity and nitrogen metabolism regulation among LAB strains [29].

#### 3.2.3. Total Phenol Content (TPC) Analysis

Monitoring phenolic compounds during fermentation is critical as they contribute to antioxidant activity and bioactivity, which may be altered by microbial metabolism [34]. As presented in Figure 2C and Table S1, the TPC of fermented QJ by all three LAB strains was significantly lower than those in control (non-fermented QJ) (*p* =0.0011, η^2^ = 0.854), decreasing from 3.29 ± 0.11 to 2.89 ± 0.03, 2.96 ± 0.08, and 3.00 ± 0.07 mg GAE/mL by the fermentation using the Lf14, Lr18 and Lp808 strains, respectively. The reduction in TPC may be caused by the enzymatic hydrolysis of phenolic substances into free form or the combination or adsorption of phenolic substances with proteins [35,36]. This finding was consistent with the results of An et al. [32], indicating that LAB utilizes nutritional resources to generate new metabolites during the fermentation process and alters the TPC of fermented QJ. Notably, Lp808-fermented QJ showed the least reduction in TPC (8.81%) compared to other LAB strains (10.24% for Lr18 and 12.28% for Lf14). It might be because *Lactiplantibacillus plantarum* has a stronger glycosidase activity, which can deglycosylate more glycosylated phenolic substances in QJ during the fermentation process, thereby releasing phenolic compounds to counteract part of the degradation effect [37,38].

#### 3.2.4. Total Flavonoid Content (TFC) Analysis

Flavonoid quantification is essential as these compounds mediate antioxidant and anti-inflammatory effects [39], which can be changed during LAB fermentation through biotransformation. The TFC of QJ exhibited significant reductions after fermentation (*p* = 0.0004, η^2^ = 0.886), decreasing from 2.89 ± 0.36 to 2.00 ± 0.12, 1.19 ± 0.24, and 1.52 ± 0.33 mg RE/mL by the fermentation using the Lf14, Lr18 and Lp808 strains, respectively (Figure 2D and Table S1). Similar results were also observed in other types of LAB-fermented plant matrices, such as hawthorn juice [40], orange juice [36] and strawberry juice [41]. The reduction in TFC during the fermentation process likely stems from the deglycosylation of enzymes [42,43]. This degradation indicated that LAB may have the ability to utilize and metabolize flavonoids to enhance their absorption and bioavailability, and further exerting physiological regulatory effects [41,44].

### 3.3. Analysis of Organic Acids

Organic acids are critical components in fermented beverages, influencing sensory properties, chemical stability, and microbial preservation [45]. Fermentation with three LAB strains significantly altered the organic acid composition of QJ (Table 1). All three LAB-fermented QJ exhibited a marked increase in lactic acid and acetic acid, concurrent with a sharp decline in succinic acid (*p* < 0.05). These shifts were attributed to LAB metabolic pathways: lactic acid accumulation reflects homofermentative carbohydrate metabolism, while acetic acid production may arise from heterofermentative metabolism or citrate utilization [46,47,48]. The decline in succinic acid likely reflects its conversion into intermediates of the tricarboxylic acid cycle or consumption by LAB under anaerobic conditions [49]. Similar results have been observed when LAB were added to apple juice [50] and orange juice [36,51].

Lf14-fermented QJ exhibited the highest lactic acid content (53.23 ± 1.03 mmol/L), whereas Lp808-fermented QJ had the most acetic acid (29.09 ± 0.50 mmol/L), suggesting differences in enzymatic activity or substrate preferences. It is worth noting that the unique increase in malic acid in Lr18-fermented QJ may be due to the malolactic enzyme activity. This is consistent with previous studies that *Lactobacillus reuteri* accumulates more malic acid during the fermentation process compared to *Lactobacillus plantarum* [52]. Such divergence aligns with studies highlighting strain-dependent metabolic traits in LAB [53,54]. In addition, the QJ fermented by all three LAB strains contained a small amount of fumaric acid, which may be the product of LAB metabolism in the presence of limited oxygen. The total acid contents increased after fermentation (49.48 mmol/L to 102.63–105.38 mmol/L). Consistent with typical LAB fermentation patterns, sugar content significantly decreased, concomitant with an increase in organic acids, likely attributed to the catabolic conversion of carbohydrates into lactic acid and other metabolites [55]. Collectively, the strain-specific acid profiles underscore the necessity of selecting tailored starter cultures to achieve desired sensory and functional outcomes.

### 3.4. Analysis of Antioxidant Capacity

**Figure 3:**
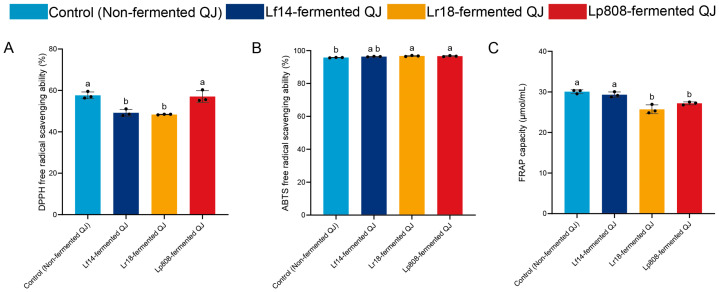
Changes in the DPPH radical scavenging activity (**A**), ABTS radical scavenging activity (**B**) and ferric reducing antioxidant power (FRAP) (**C**) of control (non-fermented QJ) and all three LAB-fermented QJ. Letters were used to indicate statistical significance between samples (*p* < 0.05) based on one-way ANOVA.

The antioxidant activity of the fermented QJ by three LAB strains was evaluated using DPPH, ABTS and FRAP methods. These assays collectively assess free radical neutralization and reducing capacity [56]. As shown in Figure 3A,C, slight decreases in DPPH scavenging activity and FRAP values were observed in Lf14, Lr18 and Lp808-fermented QJ compared to control (non-fermented QJ), likely linked to the transformation or degradation of bioactive substances such as phenolic compounds in QJ during fermentation [57,58]. Conversely, all fermented samples exhibited a marginal increase in ABTS scavenging rates by 0.6% (Lf14-fermented QJ), 1.0% (Lr18-fermented QJ), and 0.9% (Lp808-fermented QJ) compared to control (Non-fermented QJ) (Figure 3B), possibly reflecting the production of small-molecule antioxidants (e.g., peptides or organic acids) that preferentially neutralize ABTS radicals [55].

The discrepancy between the trends in ABTS versus DPPH and FRAP assays can be attributed to their differential sensitivity to various antioxidant compounds. The decline in DPPH and FRAP activities aligns with the observed significant reductions in total phenolic and flavonoid contents (Section 3.2.3 and Section 3.2.4), as these assays are particularly responsive to the electron-donating capacity of polyphenols. In contrast, the increase in ABTS scavenging activity is likely driven by the profound changes in the organic acid profile (Table 1). All fermented samples exhibited marked increases in lactic acid (from 1.53 to 49.77–53.23 mmol/L) and acetic acid (from 4.42 to 17.72–29.09 mmol/L). These small, hydrophilic organic acids are known to effectively scavenge the soluble ABTS^+^ cation, but contribute less to the reduction of the DPPH radical or the Fe^3+^-TPTZ complex in the FRAP assay. Similar findings were reported by Wu et al. [59], where LAB fermentation of apple juice altered antioxidant profiles depending on enzymatic activity and substrate utilization. This aligns with studies indicating that microbial metabolism can modify the structure of bioactive substances, thereby altering their redox properties [60]. Furthermore, we observed that the strain-specific differences in polyphenol and antioxidant retention resemble the variability reported for pre-treated oils, where enzymatic and acidic conditions modulated phenolic stability and antioxidant capacity [61].

### 3.5. Analysis of Bionic Sensory

#### 3.5.1. E-Tongue Analysis

**Figure 4:**
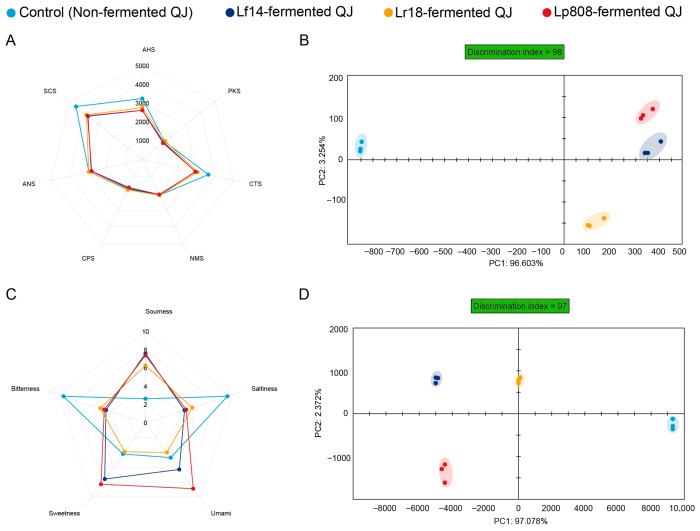
E-tongue sensor response value radar chart (**A**), E-tongue principal component analysis (**B**), taste radar chart (**C**) and E-nose principal component analysis (**D**) of control (non-fermented QJ) and all three LAB-fermented QJ.

E-tongue and E-nose are advanced biomimetic technologies designed to objectively evaluate the flavor quality of food products by simulating human olfactory and gustatory systems [62]. The E-tongue system revealed distinct taste attributes among four samples through seven sensors response analysis. Sensors AHS (sourness), CTS (saltiness), NMS (umami), ANS (sweetness), and SCS (bitterness) exhibited high response values (>2000), with AHS, CTS, and SCS demonstrating significant variability (Figure 4A). Principal component analysis (PCA) further confirmed the substantial influence of LAB fermentation, as unfermented and fermented samples showed clear spatial separation (discrimination index: 98) (Figure 4B). Notably, Lf14 and Lp808-fermented QJ clustered closely, while Non and Lr18-fermented QJ exhibited relative proximity.

Taste attributes were systematically altered by LAB fermentation, Figure 4C showed the taste radar chart of four samples analyzed by the E-tongue. Fermented samples displayed elevated sourness, correlating with lactic and acetic acid accumulation, and enhanced sweetness may be due to polysaccharide hydrolysis and sweet amino acid synthesis [63]. Bitterness and saltiness were reduced in fermented samples compared to control (non-fermented QJ), may be attributed to proteolytic degradation of bitter peptides and ion-binding effects [63]. Similarly, Lao et al. [64] found that the two-step fermentation using LAB and yeast decreased the bitterness and astringency of the *Cordyceps militaris* beverage, thus improving the taste profiles of the fermented samples. Umami (NMS) intensity increased significantly, driven by umami substances release [63]. It was reported that the flavor nucleotides can be used as an outstanding umami enhancer and the sweet taste provided by 5′-AMP can suppress the bitterness and saltiness of the fermented beverages [65]. These shifts highlight LAB’s capacity to modulate pH, degrade macromolecules, and refine flavor balance through enzymatic activity.

Earlier research also reported that the difference in starter cultures resulted in difference in taste properties of samples [1,66]. For example, Qiu et al. [1] used five commercially available LAB to ferment blended edible rose and shiitake beverage, respectively, resulting in different flavor properties. The observed taste differentiation stems from strain-specific metabolic strategies. *Lactiplantibacillus plantarum* may prioritize protease-mediated substrate conversion, generating flavor precursors and mitigating bitterness. The diversity of these enzyme activities and metabolic pathways is in line with the relevant mechanisms by which LAB regulate flavor in plant substrates [67,68]. These findings underscore the potential of tailored fermentation strategies to optimize plant-based product palatability.

#### 3.5.2. E-Nose Analysis

The E-nose is an effective tool for distinguishing odor profiles, capturing comprehensive information related to volatile compounds in samples [69,70]. PCA of E-nose data (Figure 4D) demonstrated that the cumulative contribution rate of the first (PC1) and second principal components (PC2) reached 99.45%, with PC1 alone accounting for 97.08%, indicating that these components encompassed the majority of odor-related information in both fermented and unfermented plant-based beverages. A discrimination index of 97 revealed clear spatial separation between fermented and unfermented samples in the PCA plot, as well as distinct clustering among the fermented groups, highlighting divergent aroma characteristics induced by fermentation and strain-specific metabolic activities. This finding aligns with mechanisms by which LAB regulate volatile compound dynamics through strain-dependent enzymatic activities [71,72]. Overall, the PCA results demonstrated significant variations in aroma intensity among LAB-fermented beverages, underscoring the electronic nose’s efficacy in objectively identifying aroma signatures across different samples.

### 3.6. Volatile Component Analysis

#### 3.6.1. Volatile Compounds Content Analysis

**Figure 5:**
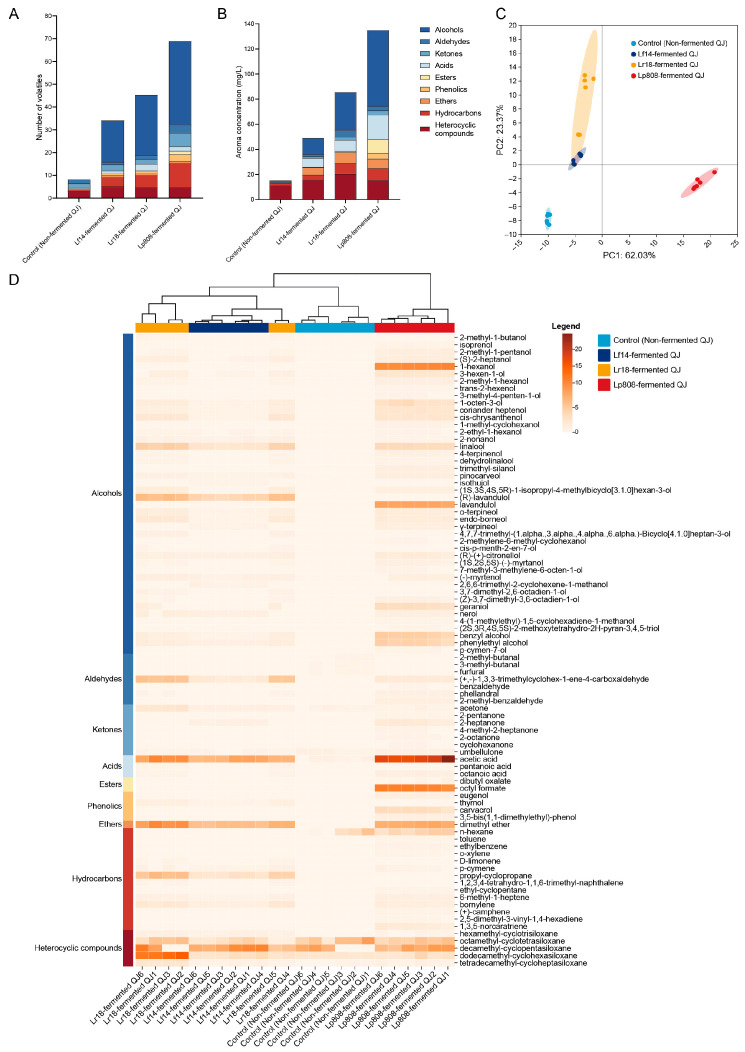
The number of volatiles (**A**), the aroma concentration (**B**), principal component analysis (**C**) and the heatmap (**D**) of control (non-fermented QJ) and all three LAB-fermented QJ. Note: The dendrogram at the top of (**D**) represents the hierarchical clustering of columns.

The volatile compound profiles of QJ fermented with three distinct LAB strains exhibited significant metabolic differences, directly impacting their final flavor attributes. Quantitative analysis via HS-SPME-GC-MS identified 87 volatile compounds (Table S2), including alcohols (44), aldehydes (7), ketones (7), acids (3), esters (2), phenols (4), ethers (1), hydrocarbons (14), and heterocyclic compounds (5) (Figure 5A,B). Alcohols dominated the volatile profiles in fermented beverages, accounting for 26–45% of total volatile content (Figure 5A,B). These alcohols, such as 1-octen-3-ol, linalool, 4-terpineol, dihydrolinalool, α-terpineol, geraniol, phenethyl alcohol, and others, are critical contributors to sensory attributes, imparting mushroom, citrus, mint, pine, rose, and earthy notes [1,14].

PCA of all detected volatiles highlighted distinct clustering patterns among control (non-fermented QJ) and fermented groups (Figure 5C). The cumulative contribution rate of PC1 and PC2 reached 85.4%, capturing most variance between fermented and control (non-fermented QJ) samples. Clear separation was observed: Non, Lf14, and Lr18-fermented QJ clustered along the negative PC1 axis, while Lp808-fermented QJ occupied the positive PC1 axis. Additionally, control (non-fermented QJ) and Lp808-fermented QJ grouped on the negative PC2 axis, whereas Lf14 and Lr18-fermented QJ aligned with the positive PC2 axis. The PCA indicated pronounced volatile differences between Lp808-fermented QJ and the other two LAB-fermented groups, alongside similarities between Lf14 and Lr18-fermented QJ.

Heatmap and hierarchical clustering analyses further delineated fermentation-driven compositional shifts (Figure 5D). Control (non-fermented QJ) exhibited elevated levels of aldehydes and ketones, which were markedly reduced after fermentation. A negative relationship between consumer acceptability and high concentrations of aldehydes has been previously described [73,74]. Conversely, fermented samples showed increased alcohols and esters, which is a well-known characteristic of LAB fermentation [75]. In addition, the enhanced production of esters and the decline of certain aldehydes after fermentation parallel earlier reports that pre-treatments such as blanching alter enzymatic activity and oxidation pathways in oils [76]. Clustering segregated control (non-fermented QJ) from all fermented groups, emphasizing fermentation-induced transformations. Among fermented beverages, Lf14 and Lr18-fermented QJ shared similarities in the types and contents of volatile substances, thus clustering together. Lp808-fermented QJ formed an independent cluster due to its unique and high content of volatile components, reflecting strain-specific metabolic activities. Notably, acetic acid was universally enriched across fermented groups, suggesting conserved carbohydrate degradation pathways in LAB. These findings demonstrate that LAB fermentation not only reduces plant-derived aldehydes but also diversifies volatile profiles in a strain-dependent manner, with phenols and alcohols serving as key discriminators.

**Figure 6:**
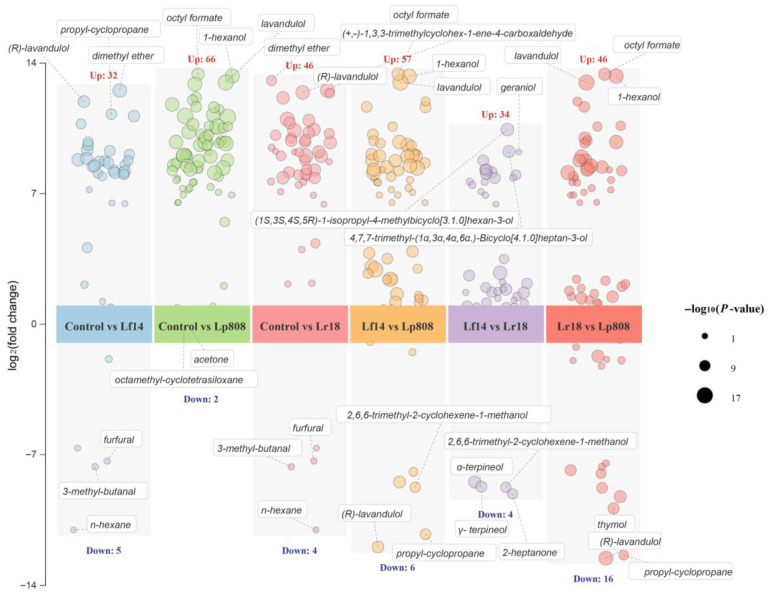
Volcano plots showing differential volatile compounds in control (non-fermented QJ) and QJ fermented by different LAB Strains. Abbreviation: Control, Control (Non-fermented QJ); Lf14, Lf14-fermented QJ; Lr18, Lr18, Lr18-fermented QJ; Lp808, Lp808-fermented QJ.

Volcano plots were employed to analyze the dynamic changes in volatile compounds in QJ induced by LAB fermentation (Figure 6). The horizontal axis represents −log_10_(*p*-value), reflecting statistical significance, while the vertical axis shows log_2_(fold change), indicating the relative content change of each compound. Point size corresponds to the degree of significance. Comparative analysis across six groups (Control vs. Lf14-, Lr18-, or Lp808-fermented QJ; and between different-strain-fermented comparisons) revealed distinct regulatory effects among LAB strains. Specifically, Lp808 exhibited the most pronounced upregulation of volatiles (66 compounds) compared to Control, including key compounds such as octyl formate and 1-hexanol. Meanwhile, in Lr18 vs. Lp808, Lp808 significantly downregulated 16 compounds, suggesting its potential in mitigating undesirable flavors. These strain-specific modulations of esters, alcohols, and aldehydes highlight the role of LAB in shaping the aroma profile and support the selection of strains for flavor enhancement in fermented QJ.

#### 3.6.2. Odor Activity Value (OAV) Analysis

The aroma profile of food products is determined by the types, quantities, concentrations, and odor thresholds of volatile compounds [69]. The OAV, defined as the ratio of a compound’s concentration to its odor threshold, is used to evaluate the contribution of individual volatile compounds to the overall aroma characteristics [77]. By analyzing the OAVs of 31 volatile compounds in fermented and unfermented QJ (Table 2), this study identified key contributors to their aroma profiles. Compounds with OAVs > 1 were predominantly alcohols. 1-Octen-3-ol exhibited the highest OAV range (123.33–970.00), imparting mushroom-like and umami characteristics [78]. Other notable alcohols included *trans*-3-hexen-1-ol (OAV: 14.50–109.50), contributing green and leafy notes [8], and geraniol (OAV: 120.00–318.00), associated with floral and fruity aromas [1]. A previous study reported an increase in these compounds in fermented edible olives inoculated with the *Lactobacillus plantarum* strain, which were obtained from linoleic acid and linolenic acid through the lipoxygenase pathway [79]. This suggests that there is a certain content of fatty acids in QJ and a similar metabolic transformation exists. Terpene alcohols such as linalool (OAV: 22.80–44.90) and α-terpineol (OAV: 13.50–73.00) enhanced citrus and woody undertones [80]. Several studies showed that these compounds, if above the threshold level, might confer desirable attributes to beverages [1,81].

In this study, Lp808 demonstrated the potential to enhance flavor characteristics in fermented QJ. Lp808-fermented QJ contained unique flavor compounds, such as octyl formate (OAV: 361.00), eugenol (OAV: 276.67), carvacrol (OAV: 428.00), and 2-methylbenzaldehyde (OAV: 30.33), which introduced distinct herbal, medicinal, and spicy notes [1]. Therefore, LAB fermentation could be a valuable strategy to enrich the aroma of MEH plant-based beverages. This discovery highlights the crucial role of fermentation agent selection in the formation of aroma.

## 4. Conclusions

This study investigated the effects of three LAB strains on the physicochemical properties, bioactivity, and flavor profiles of a plant beverage (QJ) derived from MEH. The results revealed that all three LAB strains effectively grew in the MEH plant-based matrix, achieving viable counts above 8.5 log CFU/mL. LAB fermentation notably reduced sugar content while increasing organic acids. Despite these shifts, the antioxidant capacity of QJ remained stable, potentially due to its inherent high baseline activity from raw materials. E-tongue and E-nose analysis demonstrated that fermentation significantly improved flavor characteristics, reducing bitterness and enhancing sweetness and umami. HS-SPME-GC-MS further highlighted strain-specific volatile profiles, with Lp808 uniquely producing octyl formate, eugenol, and carvacrol, contributing to a balanced aroma. Among the strains, Lp808 exhibited superior potential as a starter culture due to its dual capacity for substrate utilization and flavor modulation. These findings emphasize the importance of strain selection in tailoring flavor and functional properties of fermented MEH plant beverages. Future studies should focus on scaling these results to industrial processes and optimizing strain combinations for enhanced quality and application in MEH plant-based functional beverage production.

## Figures and Tables

**Table 1:** Contents of organic acids in control (non-fermented QJ) and three LAB-fermented QJ (mmol/L).

Organic Acid	Control (Non-Fermented QJ)	Lf14-Fermented QJ	Lr18-Fermented QJ	Lp808-Fermented QJ
Oxalic acid	6.37 ± 0.05 ^c^	6.80 ± 0.17 ^ab^	6.98 ± 0.10 ^a^	6.57 ± 0.05 ^bc^
Malic acid	12.06 ± 0.22 ^b^	8.68 ± 0.06 ^d^	14.30 ± 0.15 ^a^	9.82 ± 0.06 ^c^
Lactic acid	1.53 ± 0.01 ^c^	53.23 ± 1.03 ^a^	51.55 ± 0.39 ^a^	49.77 ± 0.80 ^b^
Acetic acid	4.42 ± 0.04 ^d^	25.19 ± 0.48 ^b^	17.72 ± 0.26 ^c^	29.09 ± 0.50 ^a^
Maleic acid	0.090 ± 0.002 ^a^	0.076 ± 0.000 ^c^	0.077 ± 0.001 ^c^	0.080 ± 0.000 ^b^
Citric acid	8.43 ± 0.73 ^b^	5.90 ± 0.78 ^c^	11.64 ± 0.95 ^a^	5.86 ± 0.17 ^c^
Succinic acid	16.58 ± 0.55 ^a^	2.73 ± 0.12 ^b^	3.08 ± 0.11 ^b^	2.76 ± 0.13 ^b^
Fumaric acid	ND	0.025 ± 0.000 ^c^	0.032 ± 0.001 ^a^	0.030 ± 0.001 ^b^
Total acids	49.48 ± 0.94 ^c^	102.63 ± 1.39 ^b^	105.38 ± 1.08 ^a^	103.98 ± 0.97 ^b^

Note: Data are expressed as mean ± standard deviation (SD), n = 3. Lowercase letters in the same row indicate statistically significant differences between samples (*p* < 0.05) based on one-way ANOVA. ND, not detected.

**Table 2:** Odor activity values of the main volatile compounds in non-fermented QJ and three LAB-fermented QJ (OVA > 1).

NO	Name	CAS	Odor Threshold (mg/kg in Water)	Control (Non-Fermented QJ)	Lf14-Fermented QJ	Lr18-Fermented QJ	Lp808-Fermented QJ	Flavor/Odor Profile
1	(S)-2-heptanol	6033-23-4	0.035	ND	5.14	28.86	36.00	Mushroom, oily, fatty, blue, cheese, moldy
2	1-hexanol	111-27-3	0.5	ND	ND	ND	20.18	Green, fruity, apple-skin, oily
3	3-hexen-1-ol	544-12-7	0.02	ND	14.50	42.00	109.50	Green, leafy
4	*trans*-2-hexenol	928-95-0	0.05	ND	ND	ND	6.80	Green, leafy, fresh, fatty, grassy, fruity, juicy
5	1-octen-3-ol	3391-86-4	0.003	ND	123.33	450.00	970.00	Mushroom, earthy, fungal, green, oily, vegetative, umami, sensation, savory, brothy
6	linalool	78-70-6	0.1	ND	22.80	44.90	35.10	Citrus, orange, lemon, floral, waxy, aldehydic, woody
7	4-terpinenol	562-74-3	0.02	ND	ND	15.50	25.50	Cooling, woody, earthy, clove, spicy, with a citrus undernote
8	(R)-lavandulol	498-16-8	0.075	ND	52.27	79.20	ND	Herbal
9	α-terpineol	98-55-5	0.02	ND	13.50	62.00	73.00	Citrus, woody, lemon, lime, soapy
10	endo-borneol	507-70-0	0.03	ND	9.67	59.00	51.33	Pine, woody, camphoreous, balsamic
11	γ-terpineol	586-81-2	0.06	ND	7.00	ND	16.83	Pine, floral, lilac
12	*cis*-p-menth-2-en-7-ol	19898-86-3	0.075	ND	ND	2.27	ND	NF
13	(-)-myrtenol	515-00-4	0.06	ND	7.83	14.17	17.50	Cooling, minty, camphoreous, green, medicinal
14	3,7-dimethyl-2,6-octadien-1-ol	624-15-7	0.03	ND	ND	14.67	ND	NF
15	geraniol	106-24-1	0.01	ND	ND	120.00	318.00	Floral, rose, waxy, fruity, peach
16	nerol	106-25-2	0.02	ND	38.50	62.00	44.00	Lemon, bitter, green, fruity, terpenic
17	benzyl alcohol	100-51-6	3.5	ND	0.11	0.32	1.30	Chemical, fruity, balsamic
18	phenylethyl alcohol	60-12-8	0.3	ND	2.10	3.97	13.77	Floral, sweet, rose, bready
19	2-methyl-butanal	96-17-3	0.0075	26.67	ND	ND	ND	Musty, rummy, nutty, cereal, caramellic, fruity
20	3-methyl-butanal	590-86-3	0.0065	46.15	ND	ND	ND	Fruity, green, chocolate, nutty, leafy, cocoa
21	furfural	98-01-1	0.03	8.00	ND	ND	ND	Brown, sweet, woody, bready, nutty, caramellic, burnt, astringent
22	2-methyl-benzaldehyde	529-20-4	0.03	ND	ND	ND	30.33	Cherry
23	2-heptanone	110-43-0	1.25	ND	0.44	ND	1.27	Cheesy, fruity, coconut, waxy, green
24	octanoic acid	124-07-2	0.2	ND	ND	1.55	3.80	Rancid, soapy, cheesy, fatty, brandy
25	octyl formate	112-32-3	0.03	ND	ND	ND	361.00	Green, oily, orange, cilantro, waxy, citrus
26	eugenol	97-53-0	0.003	ND	ND	ND	276.67	Sweet, warm, spicy, clove, phenolic, woody
27	thymol	89-83-8	0.02	ND	12.00	47.00	ND	Phenolic, medicinal, woody, spicy
28	carvacrol	499-75-2	0.0075	ND	ND	ND	428.00	Spicy, herbal, phenolic, medicinal, woody
29	3,5-bis(1,1-dimethylethyl)-phenol	1138-52-9	0.3	ND	ND	ND	1.70	NF
30	D-limonene	5989-27-5	0.03	ND	ND	8.00	ND	Sweet, orange, citrus
31	p-cymene	99-87-6	0.075	ND	3.47	8.40	5.87	Rancid, woody, citrus, spicy, pepper, bell, pepper

Note: Substances with OVA > 1 were listed in the table. The odor thresholds of the compounds here in water were obtained from the literature. Compound flavor/odor profile or type from Perflavory (http://www.perflavory.com/, accessed on 24 April 2025). ND, not detected; NF, not found.

## Data Availability

The original contributions presented in this study are included in the article/Supplementary Materials. Further inquiries can be directed to the corresponding author.

## Supplementary Materials

The following supporting information can be downloaded at: https://www.mdpi.com/article/10.3390/foods14213761/s1, Figure S1: Standard curves of soluble sugar, total plant phenol, total plant flavonoids, FRAP and organic acids.; Table S1: Complete statistical output for one-way ANOVA and Tukey’s HSD post hoc tests on biochemical composition and antioxidant capacity of QJ fermented with different LAB; Table S2: The relative contents of volatile components in non-fermented QJ and three LAB fermented QJ (mg/L).

## Abbreviations

The following abbreviations are used in this manuscript:

LAB	Lactic acid bacteria
MEH	Medicinal and edible homologous
E-tongue	Electronic tongue
E-nose	Electronic nose
HS-SPME-GC-MS	Headspace solid-phase microextraction–mass spectrometry coupled with gas chromatography–mass spectrometry
TPC	Total phenol content
TFC	Total flavonoid content
HPLC	High-performance liquid chromatography
FRAP	Ferric reducing antioxidant power
PCA	Principal component analysis
OAV	Odor activity value
