# Supplementary material for: Study on Physicochemical Properties, Antioxidant Activity and Flavor Quality in the Fermentation of a Plant-Based Beverage by Different Lactic Acid Bacteria
Source: Foods. 2025 Nov 2;14(21):3761. doi: 10.3390/foods14213761 (PMC12608928; doi:10.3390/foods14213761)
Supplement: Supplementary file 1 [file foods-14-03761-s001.zip › foods-3927429-supplementary.pdf]

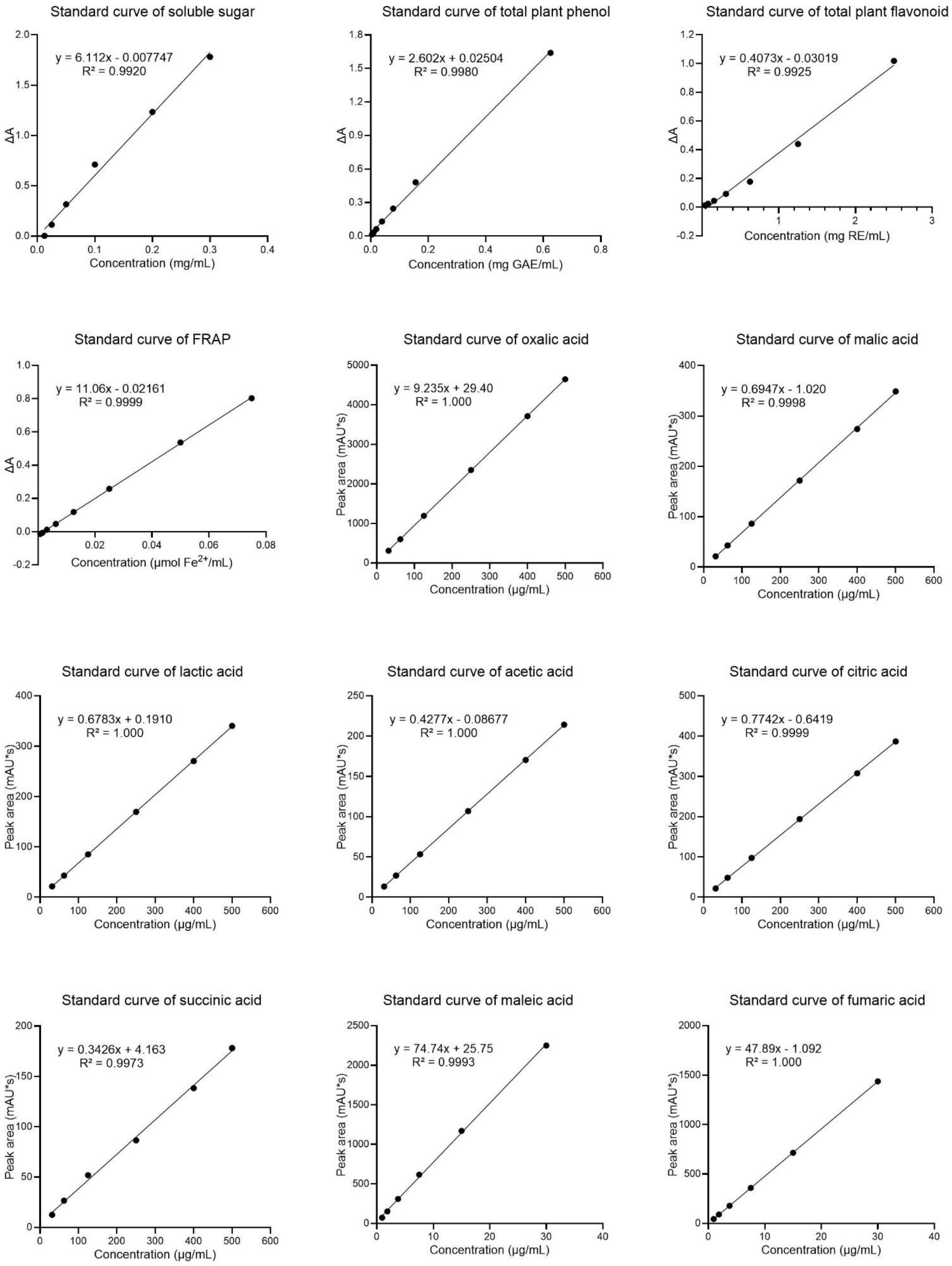

Figure S1 Standard curves of soluble sugar, total plant phenol, total plant flavonoids, FRAP and organic acids.

Table S1. Complete statistical output for one-way ANOVA and Tukey's HSD post hoc tests on biochemical composition and antioxidant capacity of QJ fermented with different LAB.

Analyte / Assay	ANOVA F-value (df)	ANOVA P-value	η² (R^2)	Comparison	Mean Difference	95% CI of Difference	Adjusted P-value
**Soluble Sugar**	*F*(3,8)=34.81	< 0.0001	0.929	Control vs. Lf14	2.637	0.032 to 5.241	0.0473
				Control vs. Lr18	7.143	4.539 to 9.748	0.0001
				Control vs. Lp808	6.617	4.012 to 9.221	0.0002
				Lf14 vs. Lr18	4.507	1.902 to 7.111	0.0024
				Lf14 vs. Lp808	3.980	1.375 to 6.585	0.0053
				Lr18 vs. Lp808	-0.527	-3.131 to 2.078	0.9135
**Total Protein**	*F*(3,8)=18.32	0.0006	0.873	Control vs. Lf14	1.760	0.846 to 2.673	0.0012
				Control vs. Lr18	1.647	0.733 to 2.560	0.0019
				Control vs. Lp808	1.762	0.849 to 2.675	0.0012
				Lf14 vs. Lr18	-0.113	-1.026 to 0.800	0.9775
				Lf14 vs. Lp808	0.002	-0.911 to 0.916	>0.9999
				Lr18 vs. Lp808	0.115	-0.798 to 1.029	0.9762
**Total Amino Acid**	*F*(3,8)=18.97	0.0005	0.877	Control vs. Lf14	-0.080	-0.827 to 0.668	0.9853
				Control vs. Lr18	0.274	-0.473 to 1.022	0.6579
				Control vs. Lp808	1.471	0.723 to 2.218	0.0011
				Lf14 vs. Lr18	0.354	-0.394 to 1.102	0.4718
				Lf14 vs. Lp808	1.550	0.803 to 2.298	0.0007

Analyte / Assay	ANOVA F-value (df)	ANOVA P-value	η^2 (R^2)	Comparison	Mean Difference	95% CI of Difference	Adjusted P-value
				Lr18 vs. Lp808	1.197	0.449 to 1.944	0.0040
**Total Phenolic Content (TPC)**	$F(3,8)=15.53$	0.0011	0.854	Control vs. Lf14	0.404	0.199 to 0.610	0.0010
				Control vs. Lr18	0.337	0.132 to 0.543	0.0034
				Control vs. Lp808	0.297	0.091 to 0.502	0.0073
				Lf14 vs. Lr18	-0.067	-0.272 to 0.138	0.7301
				Lf14 vs. Lp808	-0.108	-0.313 to 0.098	0.3928
				Lr18 vs. Lp808	-0.041	-0.246 to 0.165	0.9180
**Total Flavonoid Content (TFC)**	$F(3,8)=20.70$	0.0004	0.886	Control vs. Lf14	0.886	0.153 to 1.620	0.0199
				Control vs. Lr18	1.696	0.962 to 2.429	0.0003
				Control vs. Lp808	1.365	0.631 to 2.098	0.0015
				Lf14 vs. Lr18	0.809	0.076 to 1.543	0.0315
				Lf14 vs. Lp808	0.478	-0.255 to 1.212	0.2352
				Lr18 vs. Lp808	-0.331	-1.065 to 0.402	0.5086
**DPPH Radical Scavenging**	$F(3,8)=21.29$	0.0004	0.889	Control vs. Lf14	8.433	3.575 to 13.290	0.0024
				Control vs. Lr18	9.237	4.379 to 14.090	0.0013
				Control vs. Lp808	0.583	-4.275 to 5.441	0.9793
				Lf14 vs. Lr18	0.803	-4.055 to 5.661	0.9494

Analyte / Assay	ANOVA F-value (df)	ANOVA P-value	η^2 (R^2)	Comparison	Mean Difference	95% CI of Difference	Adjusted P-value
**ABTS Radical Scavenging**	$F(3,8)=8.995$	0.0061	0.771	Lf14 vs. Lp808	-7.850	-12.710 to -2.992	0.0037
				Lr18 vs. Lp808	-8.653	-13.510 to -3.795	0.0020
				Control vs. Lf14	-0.573	-1.237 to 0.091	0.0927
				Control vs. Lr18	-0.990	-1.654 to -0.326	0.0061
				Control vs. Lp808	-0.860	-1.524 to -0.196	0.0137
				Lf14 vs. Lr18	-0.417	-1.081 to 0.247	0.2609
				Lf14 vs. Lp808	-0.287	-0.951 to 0.377	0.5425
				Lr18 vs. Lp808	0.130	-0.534 to 0.794	0.9205
**FRAP**	$F(3,8)=23.92$	0.0002	0.900	Control vs. Lf14	0.738	-1.098 to 2.575	0.5948
				Control vs. Lr18	4.337	2.501 to 6.174	0.0003
				Control vs. Lp808	2.865	1.029 to 4.702	0.0046
				Lf14 vs. Lr18	3.599	1.762 to 5.436	0.0011
				Lf14 vs. Lp808	2.127	0.290 to 3.964	0.0247
				Lr18 vs. Lp808	-1.472	-3.309 to 0.365	0.1225

Note: Data are presented from one-way ANOVA followed by Tukey's Honestly Significant Difference (HSD) post hoc test. n = 3 biological replicates for all groups. The Brown–Forsythe test confirmed homogeneity of variances for all analyses ($p > 0.05$). η^2 (eta-squared) is calculated as the R-squared value from the ANOVA and represents the effect size. CI, confidence interval. Abbreviation: Control, Control (Non-fermented QJ); Lf14, Lf14-fermented QJ; Lr18, Lr18-fermented QJ; Lp808, Lp808-fermented QJ.

Table S2 The relative contents of volatile components in non-fermented QJ and three LAB-fermented QJ (mg/L).

No	Class	No	Name	CAS	Retention Time (RT)	Retention Indices (RI)	Control (Non-fermented QJ)	Lf14-fermented QJ	Lr18-fermented QJ	Lp808-fermented QJ	Flavor/Odor Profile	Identification method
1		1	2-methyl-1-butanol	137-32-6	14.914	1205	ND	ND	0.26±0.04^{a}	ND	Ethereal, alcoholic, fatty, greasy, cocoa, whiskey, fusel, leathery	MS, LRI
2		2	isoprenol	763-32-6	16.245	1249	ND	ND	ND	0.24±0.04^{a}	Sweet, fruity	MS, LRI
3		3	2-methyl-1-pentanol	105-30-6	17.778	1299	ND	ND	0.27±0.05^{b}	1.16±0.13^{a}	NF	MS
4		4	(S)-2-heptanol	6033-23-4	18.345	1319	ND	0.18±0.01^{c}	1.01±0.13^{b}	1.26±0.04^{a}	Mushroom, oily, fatty, blue, cheese, moldy	MS
5		5	1-hexanol	111-27-3	19.373	1356	ND	ND	ND	10.09±0.3^{a}	Green, fruity, apple-skin, oily	MS, LRI
6		6	3-hexen-1-ol	544-12-7	20.191	1385	ND	0.29±0.04^{c}	0.84±0.16^{b}	2.19±0.04^{a}	Green, leafy	MS, LRI
7		7	2-methyl-1-hexanol	624-22-6	20.541	1397	ND	ND	0.28±0.03^{b}	0.78±0.03^{a}	NF	MS
8		8	*trans*-2-hexenol	928-95-0	20.789	1407	ND	ND	ND	0.34±0.02^{a}	Green, leafy, fresh, fatty, grassy, fruity, juicy	MS, LRI
9		9	3-methyl-4-penten-1-ol	51174-44-8	20.937	1412	ND	ND	ND	0.18±0.00^{a}	NF	MS
10	Alcohols	10	1-octen-3-ol	3391-86-4	21.934	1450	ND	0.37±0.05^{c}	1.35±0.05^{b}	2.91±0.52^{a}	Mushroom, earthy, fungal, green, oily, vegetative, umami, sensation, savory, brothy	MS, LRI
11		11	coriander heptenol	1569-60-4	22.272	1463	ND	0.28±0.03^{c}	0.91±0.07^{b}	2.38±0.09^{a}	Sweet, oily, green, coriander	MS, LRI
12		12	*cis*-chrysanthenol	55722-60-6	22.532	1473	ND	0.3±0.03^{c}	2.04±0.06^{b}	2.28±0.03^{a}	NF	MS
13		13	1-methyl-cyclohexanol	590-67-0	22.89	1487	ND	ND	ND	0.44±0.02^{a}	NF	MS
14		14	2-ethyl-1-hexanol	104-76-7	22.949	1489	ND	0.17±0.01^{bc}	0.23±0.01^{a}	0.2±0.01^{ab}	Sweet, fatty, fruity	MS, LRI
15		15	2-nonanol	628-99-9	23.688	1519	ND	0.45±0.03^{b}	0.52±0.08^{a}	0.37±0.02^{c}	Waxy, soapy, musty, with, green, fruity, and, dairy, nuances	MS, LRI
16		16	linalool	78-70-6	24.417	1551	ND	2.28±0.19^{c}	4.49±0.25^{a}	3.51±0.13^{b}	Citrus, orange, lemon, floral, waxy, aldehydic, woody	MS, LRI
17		17	4-terpinenol	562-74-3	25.666	1606	ND	ND	0.31±0.02^{b}	0.51±0.03^{a}	Cooling, woody, earthy, clove, spicy, with a citrus undernote	MS, LRI
18		18	dehydrolinalool	29957-43-5	25.773	1612	ND	ND	0.27±0.02^{b}	0.4±0.05^{a}	Moldy	MS, LRI

19	19	trimethyl-silanol	1066-40-6	25.942	1621	ND	ND	ND	0.87±0.03^{a}	NF	MS
20	20	pinocarveol	5947-36-4	26.666	1661	ND	0.28±0.02^{c}	0.51±0.03^{b}	0.85±0.02^{a}	Camphoreous, woody, pine, green, thyme, fir, needle	MS, LRI
21	21	isothujol	513-23-5	26.735	1665	ND	0.35±0.02^{a}	ND	ND	NF	MS
22	22	(1S,3S,4S,5R)-1-isopropyl-4-methylbicyclo[3.1.0]hexan-3-ol	7712-79-0	26.735	1665	ND	ND	1.38±0.07^{a}	0.59±0.48^{b}	NF	MS, LRI
23	23	(R)-lavandulol	498-16-8	27.008	1680	ND	3.92±0.3^{b}	5.94±0.2^{a}	ND	Herbal	MS, LRI
24	24	lavandulol	58461-27-1	27.022	1681	ND	ND	ND	7.94±0.16^{a}	Floral, waxy, mimosa, herbal	MS, LRI
25	25	α-terpineol	98-55-5	27.36	1700	ND	0.27±0.05^{c}	1.24±0.1^{b}	1.46±0.14^{a}	Citrus, woody, lemon, lime, soapy	MS, LRI
26	26	endo-borneol	507-70-0	27.443	1705	ND	0.29±0.02^{c}	1.77±0.15^{a}	1.54±0.03^{b}	Pine, woody, camphoreous, balsamic	MS, LRI
27	27	γ-terpineol	586-81-2	27.801	1730	ND	0.42±0.04^{b}	ND	1.01±0.06^{a}	Pine, floral, lilac	MS, LRI
28	28	4,7,7-trimethyl-(1.alpha.,3.alpha.,4.alpha.,6.alpha.)-Bicyclo[4.1.0]heptan-3-ol	52486-23-4	27.806	1730	ND	ND	0.61±0.04^{a}	ND	NF	MS
29	29	2-methylene-6-methyl-cyclohexanol	1000196-32-3	27.913	1737	ND	ND	ND	0.2±0.01^{a}	NF	MS
30	30	*cis*-p-menth-2-en-7-ol	19898-86-3	28.272	1761	ND	ND	0.17±0.03^{a}	ND	NF	MS
31	31	(R)-(+)-citronellol	1117-61-9	28.358	1767	ND	0.65±0.05^{c}	0.87±0.08^{b}	1.46±0.21^{a}	Citronella, rose, leafy, oily, petal	MS
32	32	(1S,2S,5S)-(-)-myrtanol	53369-17-8	28.48	1775	ND	ND	0.24±0.02^{b}	0.55±0.05^{a}	NF	MS
33	33	7-methyl-3-methylene-6-octen-1-ol	13066-51-8	28.677	1788	ND	ND	ND	0.18±0.00^{a}	NF	MS, LRI
34	34	(-)-myrtenol	515-00-4	28.815	1798	ND	0.47±0.03^{b}	0.85±0.05^{a}	1.05±0.01^{a}	Cooling, minty, camphoreous, green, medicinal	MS, LRI

35		35	2,6,6-trimethyl-2-cyclohexene-1-methanol	6627-74-3	28.865	1801	ND	0.43±0.05[a]	ND	ND	NF	MS
36		36	3,7-dimethyl-2,6-octadien-1-ol	624-15-7	28.87	1802	ND	ND	0.44±0.05[a]	ND	NF	MS
37		37	(Z)-3,7-dimethyl-3,6-octadien-1-ol	5944-20-7	29.024	1814	ND	ND	0.2±0.02[b]	0.52±0.03[a]	NF	MS, LRI
38		38	geraniol	106-24-1	29.484	1849	ND	ND	1.2±0.2[b]	3.18±0.31[a]	Floral, rose, waxy, fruity, peach	MS, LRI
39		39	nerol	106-25-2	29.485	1849	ND	0.77±0.08[a]	1.24±0.15[a]	0.88±0.07[a]	Lemon, bitter, green, fruity, terpenic	MS, LRI
40		40	4-(1-methylethyl)-1,5-cyclohexadiene-1-methanol	19876-45-0	28.922	1806	ND	ND	ND	0.33±0.01[a]	NF	MS, LRI
41		41	(2S,3R,4S,5S)-2-methoxytetrahydro-2H-pyran-3,4,5-triol	1825-00-9	29.446	1846	ND	ND	ND	0.22±0.01[a]	NF	MS
42		42	benzyl alcohol	100-51-6	29.939	1885	ND	0.4±0.04[c]	1.12±0.14[b]	4.54±0.49[a]	Chemical, fruity, balsamic	MS, LRI
43		43	phenylethyl alcohol	60-12-8	30.387	1922	ND	0.63±0.07[c]	1.19±0.17[b]	4.13±0.53[a]	Floral, sweet, rose, bready	MS, LRI
44		44	p-cymen-7-ol	536-60-7	32.473	2114	ND	ND	ND	0.23±0.03[a]	Spicy, cumin, herbal, peppery, fatty, rue, hairy	MS, LRI
45		1	2-methyl-butanal	96-17-3	5.497	904	0.2±0.03[a]	ND	ND	ND	Musty, rummy, nutty, cereal, caramellic, fruity	MS, LRI
46		2	3-methyl-butanal	590-86-3	5.62	908	0.3±0.05[a]	ND	ND	ND	Fruity, dry, green, chocolate, nutty, leafy, cocoa	MS, LRI
47	Aldehydes	3	furfural	98-01-1	22.449	1470	0.24±0.05[a]	ND	ND	ND	Brown, sweet, woody, bready, nutty, caramellic, burnt, astringent	MS, LRI
48		4	(+,-)-1,3,3-trimethylcyclohex-1-ene-4-carboxaldehy	127128-60-3	22.724	1480	ND	1.71±0.26[b]	5.5±0.21[a]	1.67±0.11[b]	NF	MS

			de									
49		5	benzaldehyde	100-52-7	23.995	1532	ND	ND	ND	0.18±0.01^{a}	Sweet, oily, almond, cherry, nutty, woody	MS, LRI
50		6	phellandral	21391-98-0	25.413	1594	ND	ND	0.18±0^{b}	0.53±0.02^{a}	NF	MS
51		7	2-methyl-benzaldehyde	529-20-4	35.482	2406	ND	ND	ND	0.91±0.15^{a}	Cherry	MS
52		1	acetone	67-64-1	3.894	798	0.43±0.05^{b}	0.68±0.12^{b}	1.66±0.84^{a}	0.35±0.1^{b}	Solvent, ethereal, apple, pear	MS, LRI
53		2	2-pentanone	107-87-9	7.268	969	ND	ND	ND	0.3±0.04^{a}	Sweet, fruity, banana, fermented	MS, LRI
54		3	2-heptanone	110-43-0	14.143	1181	ND	0.55±0.07^{b}	ND	1.59±0.28^{a}	Cheesy, fruity, coconut, waxy, green	MS, LRI
55	Ketones	4	4-methyl-2-heptanone	6137-06-0	14.931	1206	ND	ND	ND	0.46±0.01^{a}	NF	MS, LRI
56		5	2-octanone	111-13-7	17.388	1287	ND	ND	ND	0.19±0.01^{a}	Dairy, waxy, cheesy, woody, mushroom, yeasty	MS, LRI
57		6	cyclohexanone	108-94-1	17.493	1290	ND	ND	ND	0.18±0.01^{a}	Minty, acetone	MS, LRI
58		7	umbellulone	24545-81-1	26.454	1650	0.42±0.14^{b}	0.17±0.01^{c}	0.39±0.02^{b}	0.61±0.04^{a}	Minty, pungent	MS, LRI
59		1	acetic acid	64-19-7	21.623	1438	0.5±0.08^{c}	7.17±0.88^{b}	8.45±1.57^{b}	18.67±3.03^{a}	Pungent, sour, fruit, overripe, fruit, acetic	MS, LRI
60	Acids	2	pentanoic acid	109-52-4	29.441	1846	ND	ND	0.23±0.02^{a}	ND	Acidic, dairy, milky, cheesy	MS
61		3	octanoic acid	124-07-2	31.917	2060	ND	ND	0.31±0.04^{b}	0.76±0.11^{a}	Rancid, soapy, cheesy, fatty, brandy	MS, LRI
62	Esters	1	dibutyl oxalate	2050-60-4	14.915	1205	ND	ND	ND	0.45±0.07^{a}	NF	MS
63		2	octyl formate	112-32-3	24.665	1561	ND	ND	ND	10.83±0.65^{a}	Green, oily, orange, cilantro, waxy, citrus	MS, LRI
64		1	eugenol	97-53-0	33.171	2185	ND	ND	ND	0.83±0.14^{a}	Sweet, warm, spicy, clove, phenolic, woody	MS, LRI
65	Phenolics	2	thymol	89-83-8	33.226	2191	ND	0.24±0.05^{b}	0.94±0.09^{a}	ND	Phenolic, medicinal, woody, spicy	MS, LRI
66		3	carvacrol	499-75-2	33.228	2191	ND	ND	ND	3.21±0.36^{a}	Spicy, herbal, phenolic, medicinal, woody	MS, LRI
67		4	3,5-bis(1,1-dimethylethyl)-phenol	1138-52-9	34.432	2313	ND	ND	ND	0.51±0.02^{a}	NF	MS, LRI
68	Ethers	1	dimethyl ether	115-10-6	6.182	929	ND	5.91±0.22^{c}	8.53±1.31^{a}	7.48±0.24^{b}	Ethereal	MS

69		1	n-hexane	110-54-3	2.629	634	3.14±2.2^{b}	ND	ND	3.92±0.85^{a}	NF	MS
70		2	toluene	108-88-3	9.277	1035	ND	ND	ND	0.28±0.01^{a}	Sweet	MS, LRI
71		3	ethylbenzene	100-41-4	12.168	1121	ND	ND	ND	0.43±0.02^{a}	NF	MS, LRI
72		4	o-xylene	95-47-6	12.667	1136	ND	ND	ND	0.25±0.01^{a}	Geranium	MS
73		5	D-limonene	5989-27-5	14.67	1197	ND	ND	0.24±0.01^{a}	ND	Sweet, orange, citrus, terpenic	MS
74		6	p-cymene	99-87-6	16.869	1269	ND	0.26±0.04^{a}	0.63±0.02^{a}	0.44±0.03^{a}	Terpenic, rancid, woody, citrus, spicy, pepper, bell, pepper, origanum	MS, LRI
75		7	propyl-cyclopropane	2415-72-7	19.324	1354	ND	2.45±0.29^{b}	5.33±0.92^{a}	ND	NF	MS
76	Hydrocarbons	8	1,2,3,4-tetrahydro-1,1,6-trimethyl-naphthalene	475-03-6	21.923	1450	ND	ND	0.21±0.01^{a}	ND	NF	MS
77		9	ethyl-cyclopentane	1640-89-7	22.065	1455	ND	ND	ND	0.36±0.01^{a}	NF	MS
78		10	6-methyl-1-heptene	5026-76-6	23.525	1512	ND	0.36±0.03^{c}	0.52±0.02^{b}	0.82±0.02^{a}	NF	MS
79		11	bornylene	464-17-5	23.913	1529	ND	0.87±0.13^{c}	1.95±0.13^{a}	1.12±0.15^{b}	NF	MS
80		12	(+)-camphene	5794-03-6	28.269	1761	ND	ND	ND	0.31±0.01^{a}	Minty, cooling, woody, pine, resinous, medicinal, citrus, lime, eucalyptus	MS
81		13	2,5-dimethyl-3-vinyl-1,4-hexadiene	2153-66-4	28.537	1779	ND	ND	ND	0.23±0.01^{a}	NF	MS
82		14	1,3,5-norcaratriene	4646-69-9	31.622	2032	ND	ND	ND	1.54±0.29^{a}	NF	MS
83		1	hexamethyl-cyclotrisiloxane	541-05-9	4.442	836	ND	0.44±0.07^{b}	0.59±0.13^{a}	0.23±0.05^{c}	NF	MS, LRI
84	Heterocyclic compounds	2	octamethyl-cyclotetrasiloxane	556-67-2	8.266	1004	5.29±2.22^{a}	3.08±1.04^{a}	3.61±2.42^{a}	4.82±0.91^{a}	NF	MS, LRI
85		3	decamethyl-cyclopentasiloxane	541-02-6	13.748	1169	7.61±0.85^{b}	8.82±1.69^{a}	7.73±2.92^{ab}	7.5±1.36^{ab}	NF	MS, LRI
86		4	dodecamethyl-	540-97-6	18.647	1330	2.15±0.11^{b}	2.92±0.52^{b}	9.53±5.49^{a}	2.57±0.8^{b}	NF	MS, LRI

87	5	cyclohexasiloxane tetradecamethyl-cycloheptasiloxane	107-50-6	23.088	1494	0.2±0.02[b]	0.43±0.09[b]	1.59±0.95[a]	0.41±0.13[b]	NF	MS

Note: Data are expressed as mean ± standard deviation (SD). Lowercase letters in the same row indicate statistically significant differences between samples ($p < 0.05$) based on one-way analysis of variance (ANOVA). Compound flavor/odor profile from Perflavory (http://www.perflavory.com/). ND, not detected; NF, not found.
